# Improving Thoracic Trauma Care: Locoregional Analgesia in the Intensive Care Unit

**DOI:** 10.7759/cureus.74890

**Published:** 2024-12-01

**Authors:** Guilherme Sousa, André Barbosa Ribeiro, Elena Segura-Grau, Carla Santos

**Affiliations:** 1 Anesthesiology, Unidade Local Saúde Viseu Dão-Lafões, Viseu, PRT; 2 Intensive Care Unit, Unidade Local Saúde Viseu Dão-Lafões, Viseu, PRT

**Keywords:** continuous epidural analgesia, continuous locorregional technique, erector spinal block, major trauma, multi-modality pain management, serratus plane block

## Abstract

Introduction: Pain management in thoracic trauma patients has, historically, relied heavily on systemic analgesic approaches, mostly opioids, associated with numerous adverse effects. Locoregional anesthesia/analgesia (LRAA), presents a promising alternative by specifically targeting pain pathways at the injury site.

Methods: This study investigates the impact of LRAA on pain management and clinical outcomes in thoracic trauma patients within an ICU setting. It aims to assess the effectiveness of LRAA in reducing pain and its potential to influence ICU-related outcomes. We retrospectively analyzed 43 LRAA procedures performed on 33 patients. Fourteen procedures were excluded as they were unrelated to thoracic trauma.

Results: The median age of the patients was 65 years, with a notable male predominance (84%). LRAA techniques included thoracic epidural catheters, erector spinae blocks, and serratus plane blocks. Our study found that 50% of patients who received LRAA before invasive mechanical ventilation (IMV) avoided intubation (p<0.05; odds ratio=5.3). No severe complications were associated with the catheters, despite a median utilization time of seven days. Patients who underwent LRAA before IMV had a significantly shorter ICU stay (median 9 vs. 13 days, p=0.05). The study also noted a trend toward a longer ventilation duration in patients who received LRAA before but still required IMV. In terms of mortality, there was one death in the ICU, but no 30-day post-discharge mortality. Regarding pain chronification, only 12.5% of patients experienced this issue post-discharge.

Conclusions: The study demonstrates the potential of LRAA in improving clinical outcomes for thoracic trauma patients in the ICU, particularly in reducing the need for IMV and shortening ICU stays. The findings suggest that early application of LRAA can be beneficial, although more research is needed to understand its full impact, especially on patients who still require IMV after LRAA.

## Introduction

Trauma ranks as the third leading cause of mortality, trailing only cardiovascular diseases and cancer. It is noteworthy that one in four trauma patients succumbs to the dire consequences of thoracic injuries or their associated complications [1].

Thoracic trauma spans a wide spectrum of injuries ranging from rib fractures and pneumothorax to more severe injuries, such as flail chest and pulmonary contusions. Historically, the management of pain in these patients has relied heavily on systemic analgesic approaches, most notably opioids. However, these pharmacological agents are associated with numerous adverse effects, including respiratory depression and sedation. Moreover, in patients with an already compromised respiratory function due to thoracic trauma, the use of systemic opioids can further exacerbate respiratory insufficiency and compromise pulmonary hygiene [1-3].

Consequently, the management of patients with thoracic trauma within the intensive care unit (ICU) poses a unique challenge, demanding a comprehensive approach to pain control. Not only does the intense pain associated with thoracic injuries compromise patient comfort and quality of life, but it can also significantly impact respiratory mechanics, potentially leading to life-threatening complications [1-3].

Analgesic techniques, such as locoregional anesthesia/analgesia (LRAA), present a promising complementary approach by specifically targeting pain pathways at the injury site. This approach not only appears to mitigate opioid-related complications but also fosters earlier patient mobilization, improves lung function, and reduces ventilator days. These benefits ultimately translate into improved patient outcomes and shorter ICU stays [2,3].

However, to substantiate the effectiveness of these procedures, further studies are essential to evaluate their benefits and identify potential complications. Therefore, the primary objective of this study is to assess key metrics, namely ICU length of stay, duration of invasive mechanical ventilation (IMV), the incidence of procedure-related complications, and the potential role in pain chronification.

This article was previously presented as a poster at the International Symposium on Intensive Care and Emergency Medicine, March 19-22, 2024.

## Materials and methods

**Figure 1:**
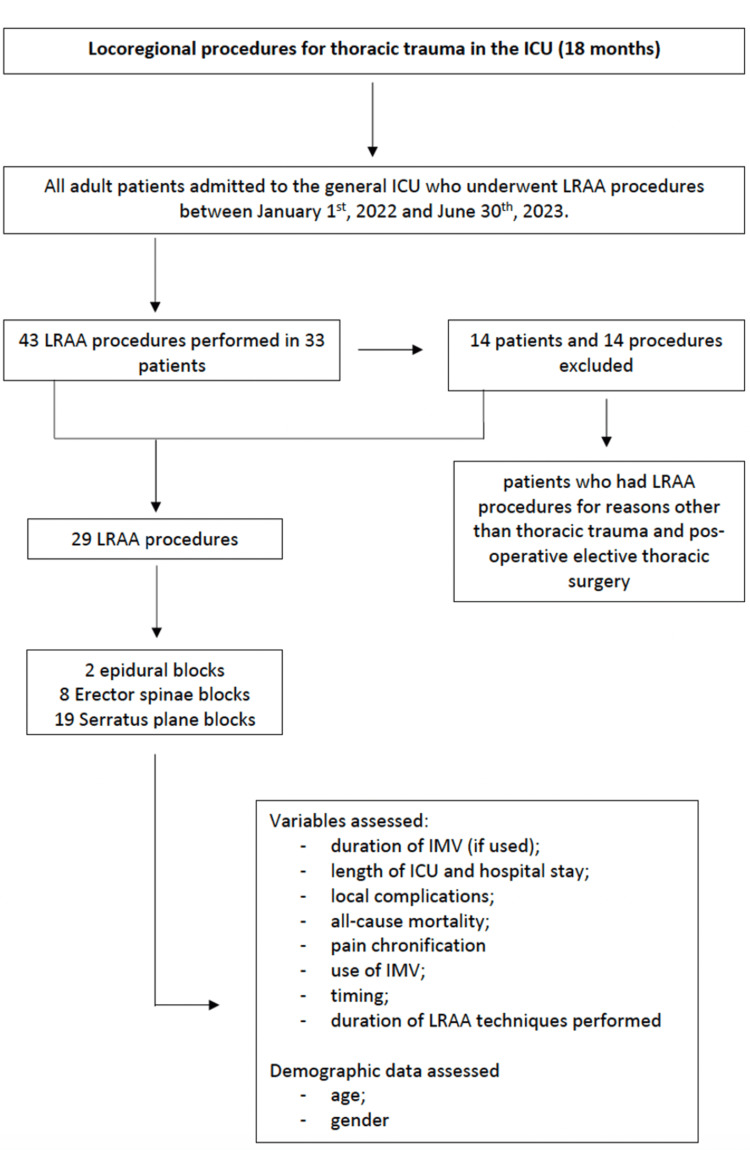
Study design ICU - Intensive Care Unit, IMV - Invasive Mechanical Ventilation, LRAA - Locoregional Anesthesia/Analgesia

We conducted a retrospective cohort study including all adult patients admitted to the general ICU who underwent LRAA procedures between January 1, 2022 and June 30, 2023. The study was conducted at the Unidade Local Saúde Viseu Dão-Lafões Hospital in Viseu, Portugal (Figure 1).

Study design and patient selection

This was a single-center, retrospective analysis. We included all adult patients (≥18 years old) admitted to our general ICU who received LRAA procedures during the 18-month study period. Patients who had LRAA procedures for reasons other than thoracic trauma and post-operative elective thoracic surgery were excluded.

Data collection

We collected data from patients' electronic medical records, including demographic information (age and gender), clinical variables (use of IMV, type, timing, and duration of LRAA techniques performed, duration of IMV (if used), length of ICU stay, length of hospital stay, local complications, all-cause mortality, and pain chronification).

To assess pain chronification and post-discharge events, we conducted telephone follow-ups with all patients. During these calls, we applied the DN4 questionnaire to estimate the probability of neuropathic pain. A DN4 score ≥4 points were considered indicative of neuropathic pain. The LRAA procedures analyzed in this study included thoracic epidural catheters, erector spinae blocks, and Serratus plane blocks.

We performed descriptive and inferential statistical analyses using SPSS version 29.0.1 (IBM Corp., Armonk, NY). Continuous variables were expressed as median and range, while categorical variables were presented as percentages. To compare outcomes between groups (e.g., patients who received LRAA before vs. after IMV), we used the Mann-Whitney U test for continuous variables (e.g., duration of IMV, length of ICU stay) due to the non-normal distribution of the data and small sample size, Fisher's exact test for categorical variables (e.g., need for intubation) given the small sample size and odds ratios with 95% confidence intervals were calculated for binary outcomes.

For the analysis of timing between trauma and LRAA application, we used the Wilcoxon signed-rank test to compare paired data. A p-value < 0.05 was considered statistically significant for all analyses. Due to the exploratory nature of this study and small sample size, we did not perform adjustments for multiple comparisons.

This study received approval from our institutional ethics board (local approval number 04/29/09/2023). Given the retrospective nature of the study and the anonymous data collection process, written informed consent was not deemed necessary. However, verbal consent was obtained from patients interviewed by telephone, during which they were informed about the study's objectives.

## Results

We analyzed a total of 43 LRAA procedures performed on 33 patients, during the study period. Fourteen procedures (14 patients) were excluded from the analysis as they were not performed in the context of thoracic trauma.

The patient cohort had a median age of 65 years (range: 21 to 80 years), with a predominant male representation (84%, n=16). The 29 LRAA procedures included two thoracic epidural catheters, eight erector spinae blocks, and 19 serratus plane blocks. Regarding timing, 68.4% of patients (n=13) underwent LRAA procedures after the initiation of IMV, while 31.6% (n=6) had LRAA procedures conducted prior to IMV if needed. Notably, 50% (n=3) of the patients in whom LRAA procedures were performed before IMV were able to avoid intubation.

For the patients who underwent LRAA procedures post-initiation of IMV, the median interval from trauma to the application of the LRAA technique was six days. On the other hand, in the group that underwent LRAA procedures before initiating IMV (if needed), the median time was lower, approximately 1.5 days (p=0.01). 

Remarkably, no infectious complications were associated with the catheters, despite a median catheter utilization time of seven days. In terms of complications associated with the technique or catheter usage, the majority (n=9) proceeded without any issues. However, there were two cases involving hematic punctures, five cases of catheter obstruction or kinking, one case of desaturation associated with lateral decubitus positioning, and two cases in which, due to the emergence of inflammatory signs near the catheter, it was preemptively removed. Additionally, there were eight cases of unintentional catheter removal, highlighting the need for secure catheter management protocols.

It is noteworthy that three out of the six patients who received LRAA procedures before IMV avoided mechanical ventilation whatsoever (p<0.05, odds ratio = 5.3). However, in those patients who required IMV despite receiving LRAA before IMV, there was a trend toward the longer duration of ventilation, despite not reaching statistical significance (median 20 days vs 8.8 days, p > 0.05), compared to patients who received LRAA procedures after IMV.

We hypothesized that patients who still required IMV despite LRAA could have more serious injuries, so we evaluated their APACHE II score, but there was no difference between these groups of patients. Interestingly, patients who received LRAA procedures before initiating IMV had a lower median duration of ICU stay (nine vs 13 days, p=0.05), compared to those patients whose LRAA procedures were performed after initiating IMV.

In terms of mortality, there was one death (5.2%) in the ICU. The 30-day mortality after hospital discharge was null. Regarding pain chronification, our study reveals a notable trend among our patients. Out of the 18 patients discharged from the ICU, two did not answer the questionnaire. Out of the 16 remaining patients, only two individuals (12.5%) experienced pain chronification.

## Discussion

The epidural block is recognized as the gold standard for managing multiple fractures associated with thoracic trauma, owing to its efficiency in providing pain relief. However, this procedure carries inherent risks and potential side effects, particularly the risk of hypotension. Promisingly, emerging techniques such as the erector spinae block and serratus plane block are gaining attention as effective alternatives. These methods show potential for providing comparable pain relief to the epidural block but with a reduced risk profile and fewer associated side effects. While these alternatives are promising, it is essential to acknowledge that there is still a lack of comprehensive studies to definitively establish their effectiveness. Ongoing research is crucial to further explore their utility in enhancing pain management for thoracic trauma patients [4].

Timing of procedure

In our study, the timing of locoregional analgesia procedures revealed that 31.6% (n=6) of patients received these procedures before IMV. Notably, an impressive 50% of the patients in whom LRAA procedures were performed before IMV successfully avoided intubation (p<0.05, odds ratio = 5.3). This phenomenon emphasizes the potential benefits of locoregional analgesia in preserving the mechanisms of spontaneous ventilation, which could contribute to improved patient outcomes and a reduced need for IMV. 

Regrettably, the majority of locoregional analgesia procedures in our study were performed after the initiation of IMV, representing 68.4% of the cases (n=13). We hypothesized that this might be due to the severity of trauma experienced by the patients and the imperative need for trauma airway protection. 

A notable difference was observed in the timing of LRAA procedures. For patients who received LRAA post-IMV initiation, the median time from trauma to LRAA application was six days. On the other hand, in the group that underwent LRAA procedures before initiating IMV (if needed), the median time was lower, approximately 1.5 days (p=0.01). This variation is likely attributed to the use of LRAA in the first group predominantly for weaning from ventilation, whereas in the latter group, the primary objective was pain relief to enhance ventilation efficiency. 

These findings suggest a potential improvement in clinical practice by hastening the application of analgesic techniques in such patients. Despite possible limitations in assessing pain, early implementation of a multimodal pain management approach is critical to prevent the chronification of pain and to provide holistic care. Additionally, the prompt initiation of these techniques could enhance ventilatory support, a key aspect in managing thoracic trauma effectively.

While our observations regarding the timing of LRAA procedures and their potential benefits on spontaneous ventilation are promising, the small cohort size emphasizes the need for larger-scale studies to further validate these trends and draw more robust conclusions.

Duration of ventilation

In our study three out of the six patients who received LRAA procedures before IMV avoided mechanical ventilation whatsoever (p<0.05, odds ratio = 5.3). However, in those patients who required IMV despite receiving LRAA before IMV, there was a trend toward the longer duration of ventilation, despite not reaching statistical significance (median 20 days vs 8.8 days, p>0.05), compared to patients who received LRAA procedures after IMV. These findings offer intriguing insights into the management of this kind of patient, particularly in the context of LRAA and the timing of IMV. 

One reasonable consideration is whether a delay in the decision-making process for initiating IMV might have occurred for these patients. Optimizing the decision-making process for intubation is vital to ensure timely intervention, particularly in cases where thoracic trauma significantly impairs respiratory function.

Another possibility to explore is whether these were polytrauma victims with more complex injuries, necessitating a prolonged period of ventilation. Furthermore, our findings raise critical questions regarding the optimal timing for intubation in patients maintaining spontaneous ventilation. Striking the right balance between preserving spontaneous breathing and preventing patient self-inflicted lung injury is a challenge. While LRAA can play a crucial role in pain management and potentially preservation of spontaneous breathing, further research is necessary to determine the ideal timing for intubation, to enhance outcomes and reduce complications. On the other hand, it is noteworthy that patients who underwent LRAA procedures before IMV experienced shorter ICU stays, highlighting the potential advantages of early pain management in thoracic trauma cases. 

These observations emphasize the multifaceted nature of decision-making in the ICU and suggest that an individualized and multidisciplinary approach to patient care is essential to achieve the best possible outcomes. Nevertheless, to comprehend these contrasting outcomes, enhance our strategies, and improve patient care in the complex setting of thoracic trauma management further evaluation is required.

Complications

Contrasting with the erector spinae and epidural blocks, which necessitate repositioning patients into the lateral decubitus position, the serratus plane block can be efficiently performed with patients in a supine position. This distinct advantage not only expedites the procedure but also eliminates the risks associated with patient movement. Our own experience highlights this advantage, as we encountered a case where the patient experienced desaturation during the repositioning process required for an epidural block. This emphasizes the potential superiority of the serratus plane block in terms of safety and practicality, making it an increasingly favored option for thoracic trauma pain management.

Understanding the catheter-specific risks within our practice is crucial. It is noteworthy that the majority of procedures in our clinical practice involve fascia blocks, which come with the distinct advantage of reducing the likelihood of post-procedure nerve injury. Nonetheless, it is essential to remain vigilant. Previous research documented bacterial colonization, leading to infection rates ranging from 0% to 3%. In a recent retrospective analysis based on a registry of cases where peripheral nerve catheters remained in place for up to 15 days, an infection rate of 2.9% was observed [5]. Remarkably, we had no infectious complications associated with the catheters, despite a median catheter utilization time of seven days. In two cases, catheters were proactively removed due to the onset of inflammatory signs, reflecting the stringent monitoring applied.

However, it is important to acknowledge that most complications encountered, such as catheter obstruction, kinking, and accidental removal, could have been averted, highlighting the importance of implementing monitoring protocols and investing in training programs for healthcare professionals handling these catheters daily. Despite these issues, our findings reinforce the safety and efficacy of locoregional thoracic analgesia procedures, while also stressing the necessity of vigilant monitoring and timely interventions to manage potential complications effectively. In order to enhance our commitment to patient safety, we proactively responded to the results of our study on catheter-specific risks. Recognizing the crucial importance of this subject, targeted awareness and training initiatives have been implemented. These actions were designed to consolidate the expertise of our healthcare team and ensure a comprehensive understanding of the risks and optimal practices associated with catheter usage. Our approach not only underscores the dedication to continuously improve but also reinforces our commitment to patient safety by translating insights from research into substantial actions that increase the quality of care provided in our service.

Pain chronification

In our study, a relatively low proportion, two individuals (12.5%), experienced pain chronification. One of these cases involves a young man who experienced a vertebro medullary trauma, resulting in paraplegia, who remains under the care of a chronic pain unit. The second case corresponds to a patient who scored four points on the DN4 questionnaire, which typically suggests neuropathic pain. He has been followed by his general practitioner, and, although an evaluation by the chronic pain unit was suggested, the patient declined, considering that his pain does not significantly impact his daily life.

These outcomes are encouraging, especially in light of literature indicating pain chronification rates in cases of chest trauma exceeding 55%. This figure, however, is subject to considerable variability, reflecting the complexities and challenges inherent in the semiotics and characterization of chronic pain [6].

Limitations

This study acknowledges several limitations. Primarily, it was conducted as a single-center retrospective analysis, which may limit the generalizability of the findings and increase susceptibility to bias. The reliability of the presented data hinges on the accuracy and completeness of data extraction from medical records. Additionally, the absence of a control group precludes direct comparisons with other analgesic techniques. However, it is noteworthy that the variables assessed were objective and not reliant on patient recall. Despite these constraints, the study offers valuable insights into the impact of LRAA on clinical outcomes in patients with thoracic trauma.

## Conclusions

In conclusion, the application of LRAA techniques in managing thoracic trauma in ICU patients presents compelling results. The intricate relationship between the timing of LRAA administration, the onset and duration of IMV, and their combined effect on clinical outcomes merits further exploration. These initial findings highlight the necessity for a thorough assessment of trauma severity and related injuries to clarify the reasons behind the observed variances in outcomes.

As understanding of LRAA's role in pain management evolves, this study contributes meaningful insights to the broader discourse on its potential impact on pain management and patient outcomes, particularly in the critical care environment of thoracic trauma management.

## References

[1] Dogrul BN, Kiliccalan I, Asci ES, Peker SC (2020). Blunt trauma related chest wall and pulmonary injuries: an overview. Chin J Traumatol.

[2] Mukherjee K, Schubl SD, Tominaga G (2023). Non-surgical management and analgesia strategies for older adults with multiple rib fractures: a systematic review, meta-analysis, and joint practice management guideline from the Eastern Association for the Surgery of Trauma and the Chest Wall Injury Society. J Trauma Acute Care Surg.

[3] Flarity K, Rhodes W, Berson A (2017). Guideline-driven care improves outcomes in patients with traumatic rib fractures. Am Surg.

[4] Chin KJ, Versyck B, Pawa A (2021). Ultrasound-guided fascial plane blocks of the chest wall: a state-of-the-art review. Anaesthesia.

[5] Capdevila X, Iohom G, Choquet O, Delaney P, Apan A (2019). Catheter use in regional anesthesia: pros and cons. Minerva Anestesiol.

[6] Kahloul M, Kacem I, Sboui MM (2020). Chronic pain following chest trauma: prevalence, associated factors, and psychosocial impact. Pain Res Manag.

